# Dark stains on rock surfaces in Driny Cave (Little Carpathian Mountains, Slovakia)

**DOI:** 10.1007/s00792-016-0853-7

**Published:** 2016-06-17

**Authors:** Rafał Ogórek, Mariusz Dyląg, Bartosz Kozak

**Affiliations:** 1 Department of Genetics, Institute of Genetics and Microbiology, University of Wroclaw, Przybyszewskiego Street 63/77, 51-148 Wroclaw, Poland; 2 Department of Genetics, Plant Breeding and Seed Production, Wrocław University of Environmental and Life Sciences, pl. Grunwaldzki 24a, 50-363 Wroclaw, Poland

**Keywords:** Driny Cave, Dark stains, Enzymatic activity, *Penicillium glandicola*

## Abstract

Mycobiota are important in underground ecology. In 2014, we discovered dark stains on clayey sediments on the walls of Driny Cave, Slovakia. Our description is based on the morphology of the fungus and the phylogenetic relationships of the internal transcribed spacer (ITS) region. In addition, data on its capacity for the production of extracellular enzymes, growth, and survival in vitro at different temperatures are reported. Our analyses revealed that this dark stains on the wall was produced by *Penicillium glandicola*. The fungus was able to synthesize amylases, proteases and cellulases, but not pectinases and keratinases. The vegetative structures of mycelium of this fungus are viable in vitro after storage at cool temperatures (from −72 to 5 °C), and show active growth at temperatures from 5 to 25 °C, but without spore germination, and without active growth at 30 and 37 °C. *Penicillium glandicola* is a psychrotolerant species and belong to var. *glandicola*.

## Introduction

Microorganisms play a crucial role in maintaining the delicate ecological balance of the earth, and they are capable of colonizing almost every niche (Sustr et al. 2005; Bastian et al. 2010). Therefore, the mycobiota are very important for underground ecology, because the fungi present are decomposers or parasites and probably constitute the major food source for various organisms (Sustr et al. 2005; Walochnik and Mulec 2009; Bastian et al. 2010). Generally, evidence for microbial activity in a cave includes spots on the cave surfaces, unusual coloration of speleothems, precipitates, corrosion residues, structural changes, and the presence of biofilms (Barton 2006). Additionally, microscopic fungi and bacteria may be isolated from various substrates and places in caves and other underground sites such as air, sediments, vermiculations, bat guano, decaying organic material, rock surfaces, bioaerosols, etc. (Nováková 2009; Ogórek et al. 2016a, b; Walochnik and Mulec 2009; Vanderwolf et al. 2013; Borda et al. 2014; Popović et al. 2015).

On the other hand, ecosystems, such as caves or underground facilities created by man are one of the most inhospitable habitats for microbial life (especially for fungi) due to nearly complete darkness, relatively constant low temperatures of air and water, and a poor supply of easily degradable organic matter, with the exception of beneficial relative humidity of air (Vanderwolf et al. 2013; Pusz et al. 2014, 2015; Ogórek et al. 2014a). Therefore, the majority of fungi cannot usually actively grow in the environment of underground facilities, but they are present regularly or rarely as spores or conidia, carried by water, air currents, bats, arthropods and humans (Kubátová and Dvořák 2005; Jurado et al. 2010; Vanderwolf et al. 2013; Griffin et al. 2014). The most fungi are obtained from the twilight zone or places with access to the external environment, e.g., ventilation shafts (Ogórek et al. 2013, 2014a, b, c; Ogórek and Lejman 2015).

Currently, the literature reports many cases of the visible presence of bacteria in caves, e.g., yellow subaerial biofilms on a stalactite and rock surfaces (Jurado et al. 2010, Mulec et al. 2015) or yellow and white microbial mats on rock surfaces (Northup et al. 2011), and many others (Barton and Northup 2007; Ivanova et al. 2013; Marshall Hathaway et al. 2014). However, similar reports with regard to the active growth of filamentous fungi on rock surfaces in caves are very few and mostly relate to the Lascaux Cave in France (Martin-Sanchez et al. 2011, 2012a, b; Saiz-Jimenez et al. 2012).

Visible growth of fungi in caves more often was found on wood, carcasses, bat droppings (e.g. *Trichoderma polysporum*), and entomopathogenic fungi have been found on dead or hibernating insects (Kubátová and Dvořák 2005; Nováková 2005, 2009). Additionally, it should be noted that the active growth of fungi in buildings are frequently reported, and they are quite common, especially under conditions of poor ventilation and high moisture, but on walls in caves they are exceptional (Garg et al. 1995; Berner et al. 1997; Gorbushina et al. 2004).

The aim of this study was to perform molecular and morphological identification of fungi isolated from the dark stains on clayey sediments on the wall in Driny Cave. Additionally, the isolated fungus was also assessed for the production of extracellular enzymes, growth rates and survival at different temperatures.

## Materials and methods

### Study area

Driny Cave is located in the Smolenice Karst in the Lesser Carpathian Mountains, south-west from Smolenice, in the Trnava district and near the recreation resort Jahodník (Slovakia). Geographic coordinates of the cave are 48°50′04″N, 17°40′20″E. Its entrance is situated on the western slope of Driny Hill at 399 m a.s.l. and its length is 680 m. The cave was discovered in 1929 by Jan Banic and Jan Vajszabel, and it was opened to the public in 1935 with provisional electric lighting 175 m in length. Now, the length of the tourist path is 410 m (Lehotská et al. 2011; Briestenskỳ et al. 2011). The air temperature in the cave is between 7.1 and 7.8 °C, and relative humidity is between 92 and 97 % (Bella et al. 2001). In 2014 alone Driny Cave was visited by ca. 31 859 people (Nudziková 2014).

### Sample collection

**Fig. 1:**
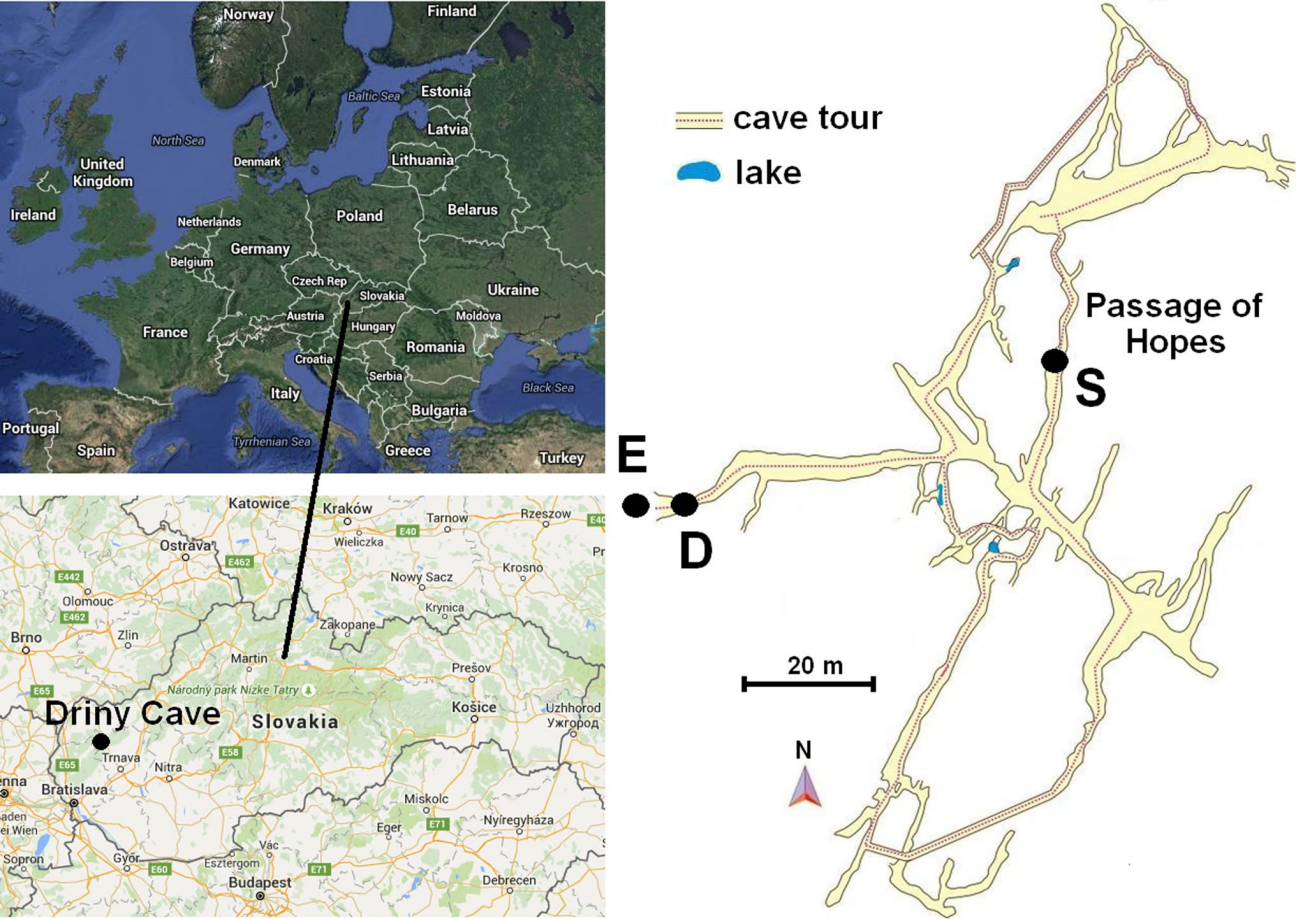
Geographic location of Driny Cave in Slovakia and map of the tourist route: *E* entrance and exit of the cave, *D* airlock door, *S* sampling location

**Fig. 2:**
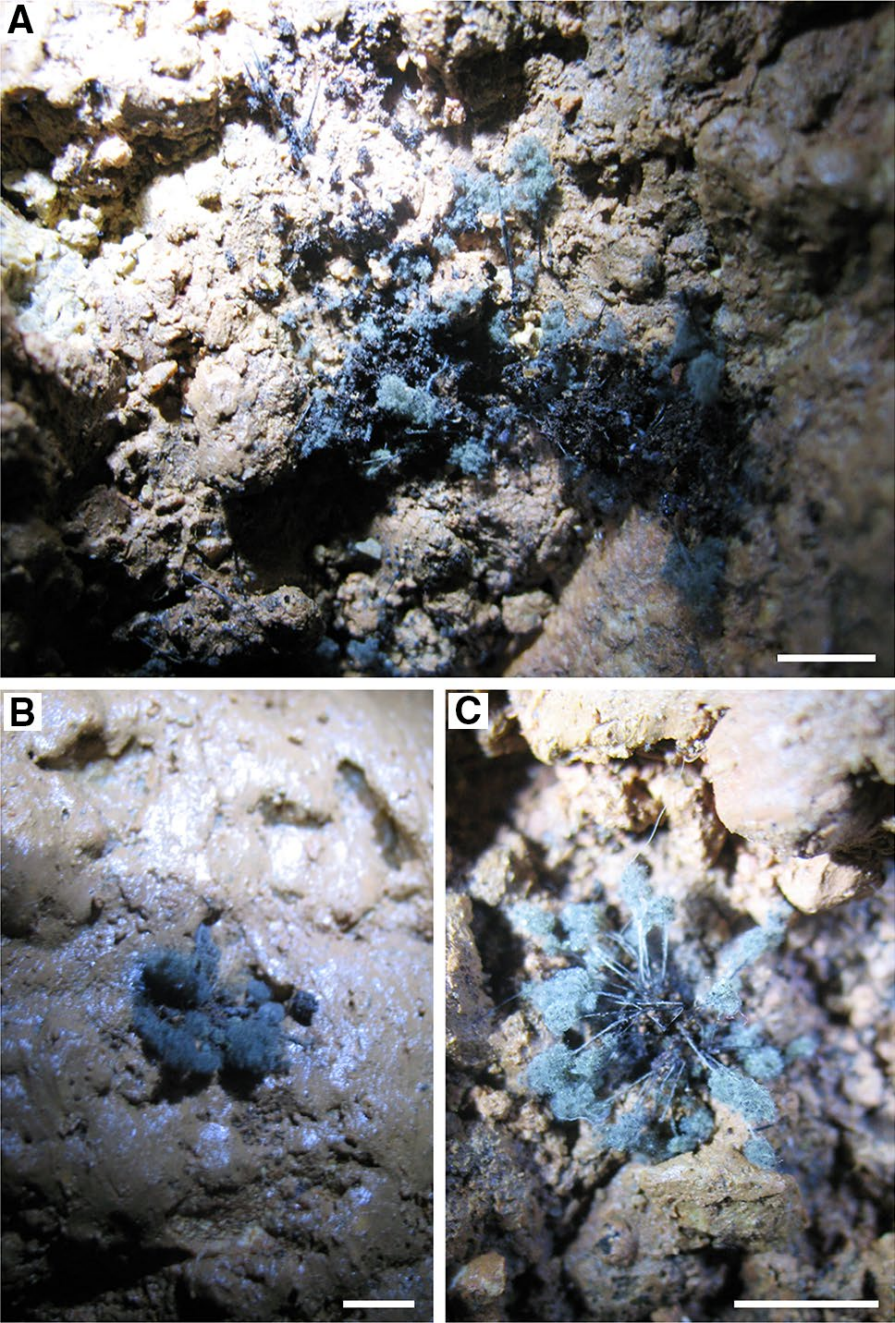
Dark stains on clayey sediments on the walls in Driny Cave—coremia of *Penicillium glandicola*. *Scale bars* *A* 20 mm; *B*, *C* 4 mm

The samples were taken on 25 July 2014. The dark stains was found only in a single location on clayey sediments on the wall (about 1.0 m above the level of the floor) in the cave (Passage of Hopes)—Figs. 1 and 2. The samples were collected using sterile swabs wetted in physiological saline (0.85 % NaCl), in transport tubes. Material was sampled with nine swabs.

### Isolation of fungi from swabs (streaking, rinse procedure and putting swabs on medium)

Streaking procedure*—*the surface of solidified Potato Dextrose Agar medium (PDA, Biocorp) was streaked in a zigzag pattern with a swab (in three replicates). Rinse procedure—sample was shaken for 20 min in a 50-ml Erlenmeyer flask containing 10 ml of sterile distilled water. After shaking, the sample was placed in a Petri dish, on solidified PDA medium (in three replicates), using the serial dilution technique. Putting swabs on medium—sample was placed on Petri dishes with solidified PDA medium (in three replicates).

### Identification of fungi

After incubation of the samples from swabs (8 and 25 °C, 7–28 days), emerging colonies of fungi on the plates were subcultured on PDA media (isolates were purified by the single spore method) for morphological and molecular identification.

**Fig. 3:**
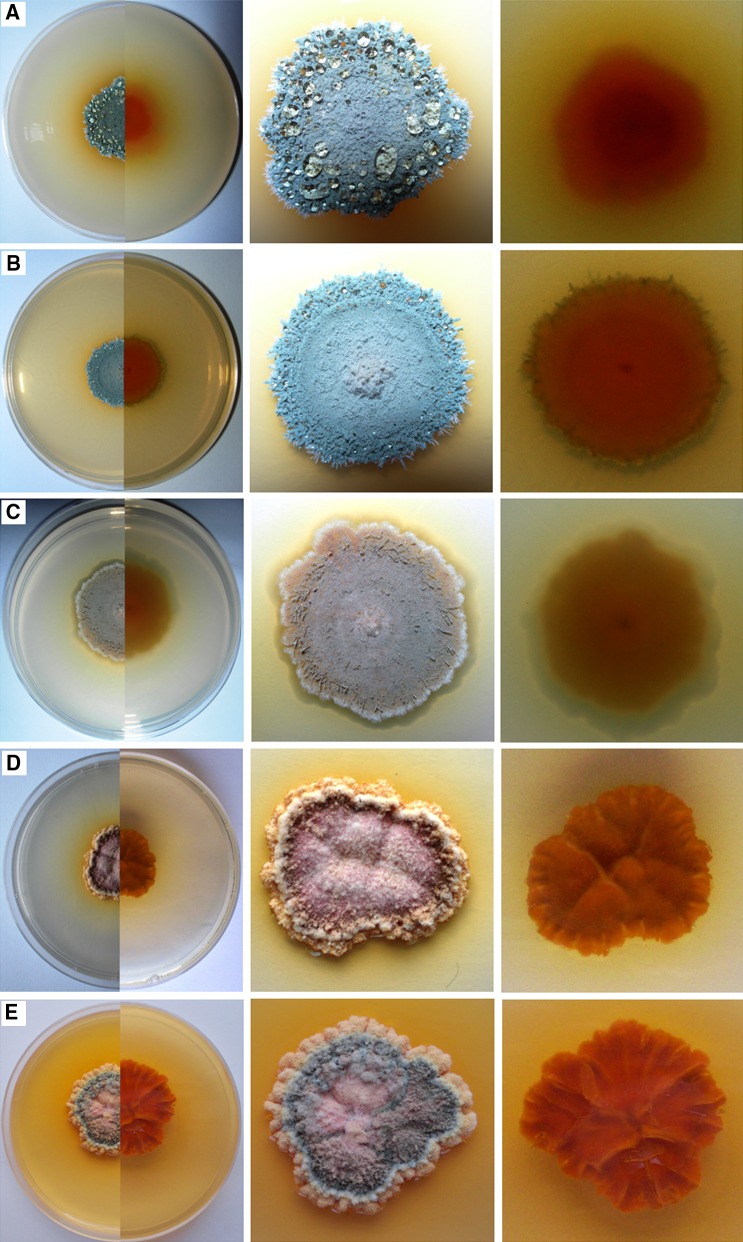
*Penicillium glandicola*, 10-day-old culture at 25 °C, *top* and *bottom* view of a colony on media: **a** PDA; **b** MEA; **c** Czapek-Dox Agar; **d** CYA; **e** YPG

**Fig. 4:**
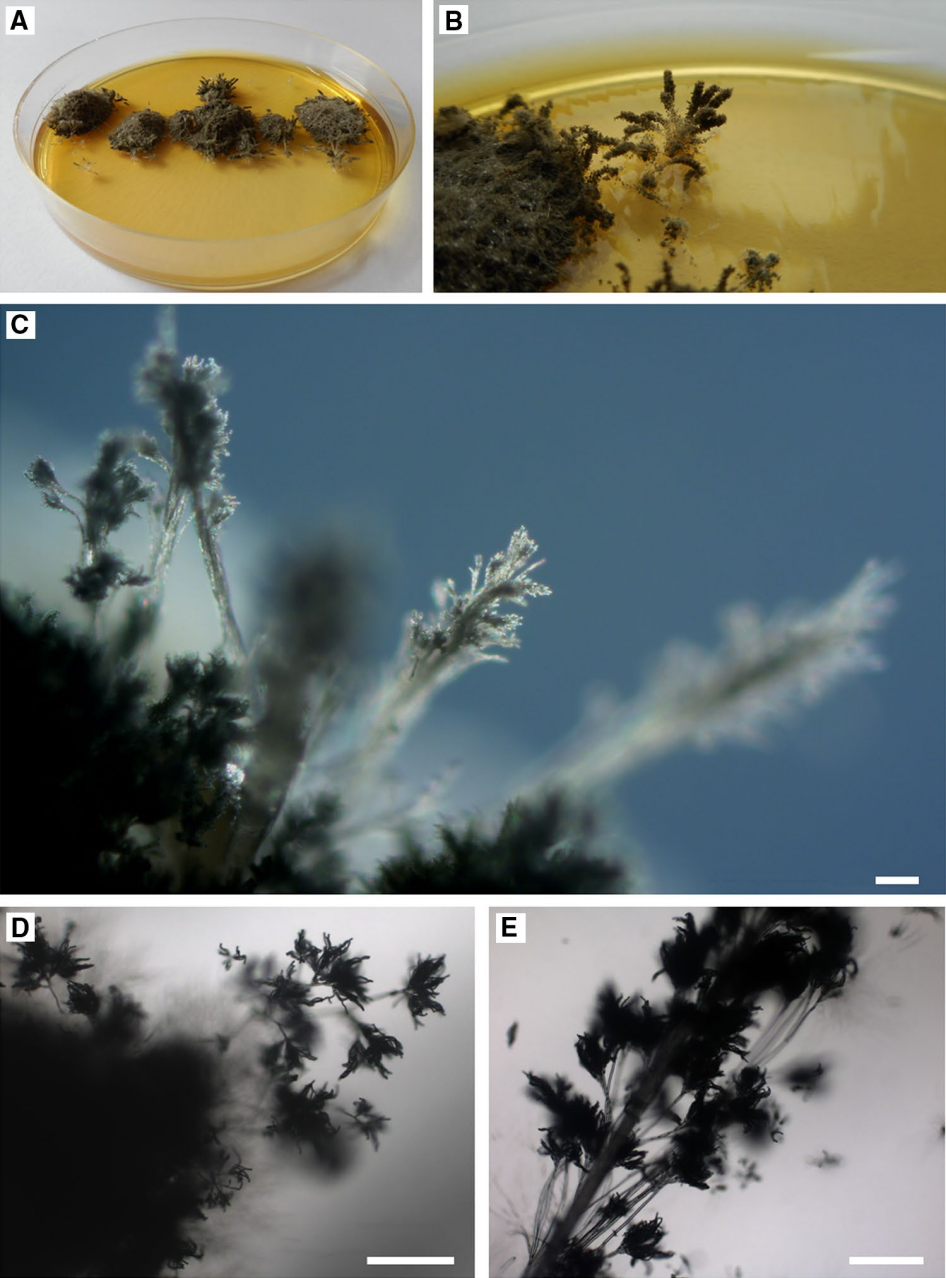
*Penicillium glandicola* on PDA, 28-day-old culture at 8 °C: **a**, **b** top view of culture (after isolation of fungi from swabs—rinse procedure); **c**, **d**, **e** Petri dish cultures under the optical microscope, branched conidiophores. *Scale bars* 200 μm

The morphological identification of the collected fungi was performed using macro- and microscopic observations of the colonies that had grown on the culture media: PDA, Malt Extract Agar (MEA, Biocorp), Czapek-Dox Agar (1.2 % agar, Biocorp), CYA (Czapek Yeast Autolysate agar: 30.0 g L^−1^ sucrose, 15 g L^−1^ agar, 5.0 g L^−1^ yeast extract, 3.0 g L^−1^ NaNO_3_, 1.0 g L^−1^ K_2_HPO_4_, 0.5 g L^−1^ KCl, 0.5 g L^−1^ MgSO_4_·7H_2_O, 0.01 g L^−1^ FeSO_4_·7H_2_O), and YPG (yeast extract peptone glucose: 10.0 g L^−1^ yeast extract, 20.0 g L^−1^ peptone, 20.0 g L^−1^ glucose, 15.0 g L^−1^ agar)—Fig. 3. Plates were incubated in plastic boxes for 7 day in the dark at 25 °C (Fig. 4). The fungi were identified using diagnostic keys (Seifert and Samson 1986; Pitt and Cruickshank 1990; Visagie et al. 2014). Subsequently, the fungus was genetically analyzed to confirm the affiliation of the species.

DNA was extracted from 10-day-old strains on PDA medium, according to the CTAB method (Doyle and Doyle 1987) with minor modifications (Ogórek et al. 2012). Amplification of DNA was performed in a 50 μl reaction mixture using the 2 × PCR mixture containing a *Taq* polymerase (0.1 U µL^−1^), dNTP mix (2 mM), MgCl_2_ (4 mM) (Blirt), 0.25 μM of each primer, ITS1: TCCGTAGGTGAACCTGCGG, ITS4: TCCTCCGCTTATTGATATGC, and ITS1 and ITS4 primers, ITS1: TCCGTAGGTGAACCTGCGG, ITS4: TCCTCCGCTTATTGATATGC (White et al. 1990) and 45 ng of genomic DNA. Amplification was performed in the Biometra thermal cycler for 35 cycles. After initial denaturation for 5 min at 94 °C, each cycle comprised 30 s denaturation at 94 °C, 30 s annealing at 55 °C, 45 s extension at 72 °C with a final extension for 7 min at 72 °C at the end of 35 cycles. The amplification product was separated on agarose gel (1.5 %), visualized by UV light, purified from gel and sequenced by the Sequencing Service at Macrogen (http://dna.macrogen.com/eng/). The PCR product sequences were compared with the published ITS sequence database from the National Center for Biotechnology Information (NCBI, Bethesda, MD, USA) using the BLAST algorithm (http://www.ncbi.nlm.nih.gov/).

### Enzymatic activity

The fungal strain was screened for the production of extracellular amylases, proteases, cellulases and pectinases by plate assay according to Ogórek (2016).

Additionally, it was assessed for the production of keratinophilic enzymes using the in vitro hair perforation test. Cut human blonde hairs (pieces ca. 2 cm, free from dust, oil and dandruff) from a 2.5-year-old child were thoroughly washed and rinsed in distilled water. After drying at room temperature, ca. 30 hair segments were placed into a glass Petri dish and sterilized in an autoclave (1 atm. for 20 min). After the Petri dishes had cooled, 25 ml of sterile distilled water and 100 µL of a sterile 10 % yeast extract solution were aseptically added to each plate and inoculated with the investigated culture (spore suspension, prepared from 10-day-old colonies). The positive control was also prepared in a similar way, but with *Trichophyton mentagrophytes* (clinical strain from Dyląg M. collection, Department of Genetics, Institute of Genetics and Microbiology, University of Wroclaw) and the negative control without fungi. All variants of experiments were incubated at 25 °C for 4 weeks. Single hairs or their segments were removed from the Petri dishes, and cleared in sterile distilled water. Then, an individual hair was placed on the microscopic slide and stained with lactophenol for microscopic observations. This allowed us to detect whether any hair perforation had taken place compared to the positive control with *T. mentagrophytes*.

### Survival at different temperatures

Fourteen-day-old cultures on PDA slant and YPG slant were placed in different temperatures: −72, −25, −10, and 5 °C. After 14, 28, 42, and 56 days of storage at each temperature, the fungal inocula from three slants for each medium were placed mycelium down in the center of a Petri dish, on the PDA and YPG media, in three replicates. The survival of fungus (growth or no growth) was evaluated after 10 days incubation in the dark at 25 °C.

### Growth at different temperatures

Fungal spores were obtained from 14-day-old cultures, which were cultivated on PDA slant, and spores were suspended in a solution of 0.85 % NaCl with 0.025 % Tween 80. The spore suspensions (OD_600_ = 0.125, ~0.5 McFarland) were placed (5 µL) in the center of a Petri dish, on the PDA and YPG media (three replicates for each medium). Then, they were placed at different temperatures: 5, 10, 15, 20, 25, 30, and 37 °C. After 2, 4, 6, 8, and 10 days of inoculation in the dark, the colony growth was measured using an electronic digital caliper.

## Results

**Fig. 5:**
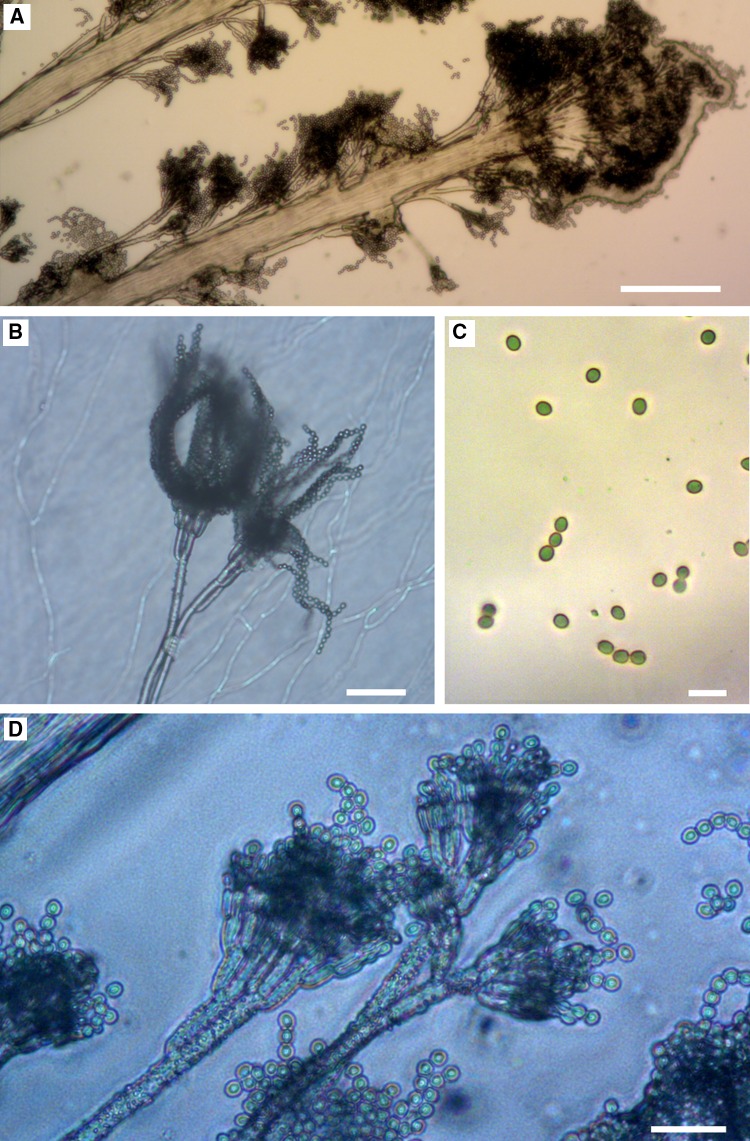
*Penicillium glandicola* under the optical microscope, 7-day-old culture at 25 °C: **a** branched conidiophores (PDA); **b** branch, ramus, metulae, phialides and conidia (MEA); **c** subglobose to ellipsoidal conidia (PDA); **d** stipes of rough branched conidiophores (PDA). *Scale bars* *A* 200 μm; *B*, *D* 20 μm; *C* 10 μm

We found active growth of fungi coating the surface of the clayey sediments on the wall in Driny Cave—Figs. 1 and 2. The swabs contained only one fungal species: *Penicillium glandicola* (Oudem.) Seifert & Samson (Figs. 2, 3, 4, 5; Table 1).

**Table 1:** BLAST analysis (the sequences were compared to *Penicillium glandicola* strain FRR 2036, Accession AY373916), and survival at different temperatures of fungus growing on the clayey sediments on the wall in Driny Cave

Accession	Amplificaton product size (bp)	Query cover (%)	Identities (%)	*E* value
KU687324	538.0	98.0	99.0	0.0

Incubation period (day)	5.0 °C	−10.0 °C	−25.0 °C	−72.0 °C
Survival at different temperatures				
14	+	+	+	+
28	+	+	+	+
42	+	+	+	+
56	+	+	+	+

“+” fungal growth after a given period of storage on slant at a given temperature

The identification with a diagnostic key was confirmed by BLAST analysis of ITS1, 5.8S and ITS2 regions amplified in PCR (Table 1). The appearance of the colony of this species was determined by growth on different culture media, especially the color of aerial mycelium and rate of growth. It usually secreted a yellow to brown soluble pigment into the media. Additionally, it was distinguished by a granular texture from fasciculate to coremiform (especially on CYA and YPG media), usually with small coremia apparent at the margins. On CYA and YPG media, the reverse side of the colony showed furrows radially and was usually deep brown—Fig. 3. This species formed rough stipes with fasciculate texture, penicilli terverticillate to quaterverticillate, metulae sometimes apically inflated, phialides very short and subglobose to ellipsoidal, and conidia yellow-green in the mass with smooth walls (2.95 × 3.90 μm)—Figs. 4 and 5.

**Fig. 6:**
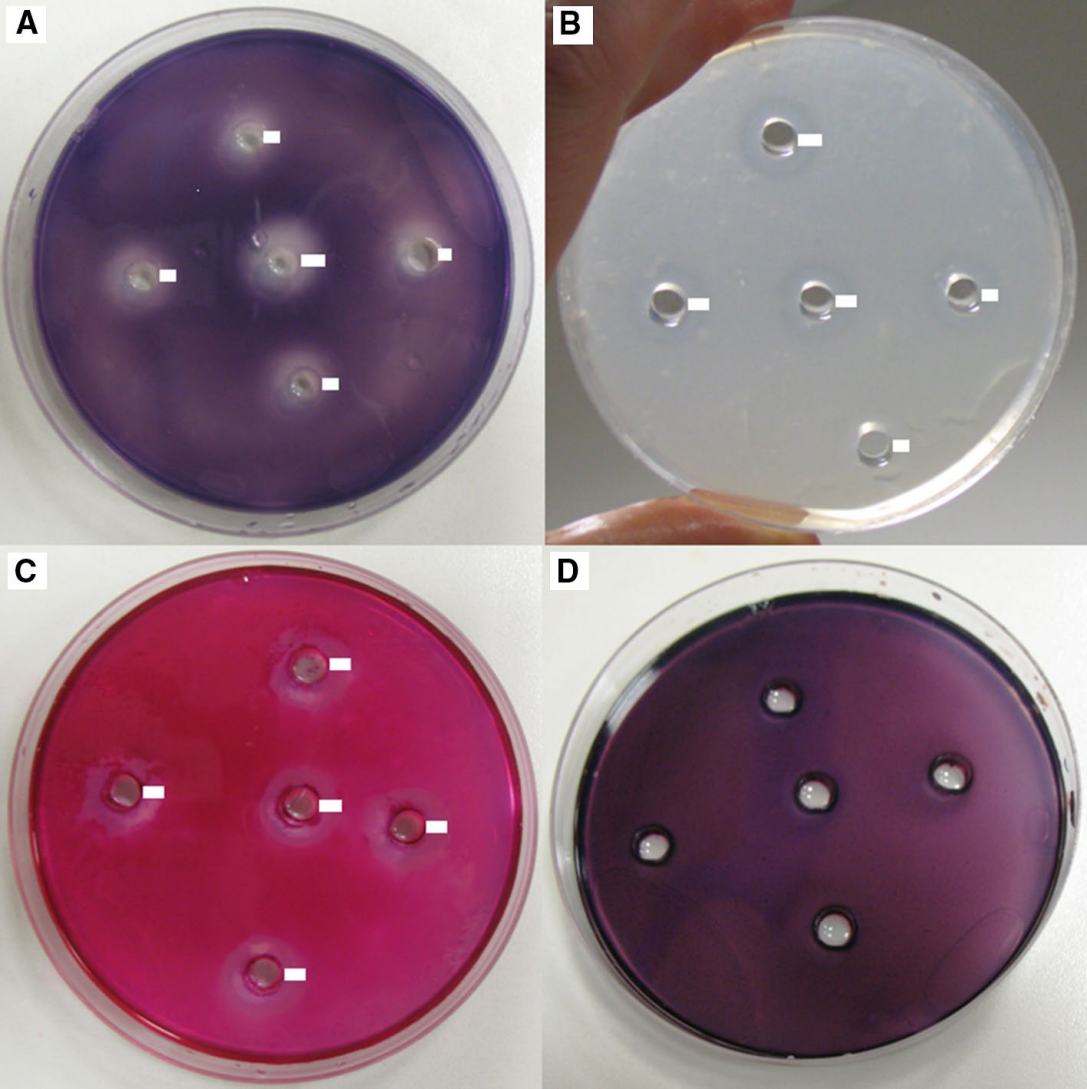
Detection of extracellular enzyme activities in *Penicillium glandicola* by plate assay: **a** amylolytic activity; **b** proteolytic activity; **c** cellulolytic activity; **d** pectinolytic activity not detected. Average enzymatic units (EU) synthesized by fungi in 1 mL of medium 3.54 × 10^−3^ EU for amylases, 1.37 × 10^−3^ EU for proteases and 3.10 × 10^−5^ EU for cellulases

**Fig. 7:**
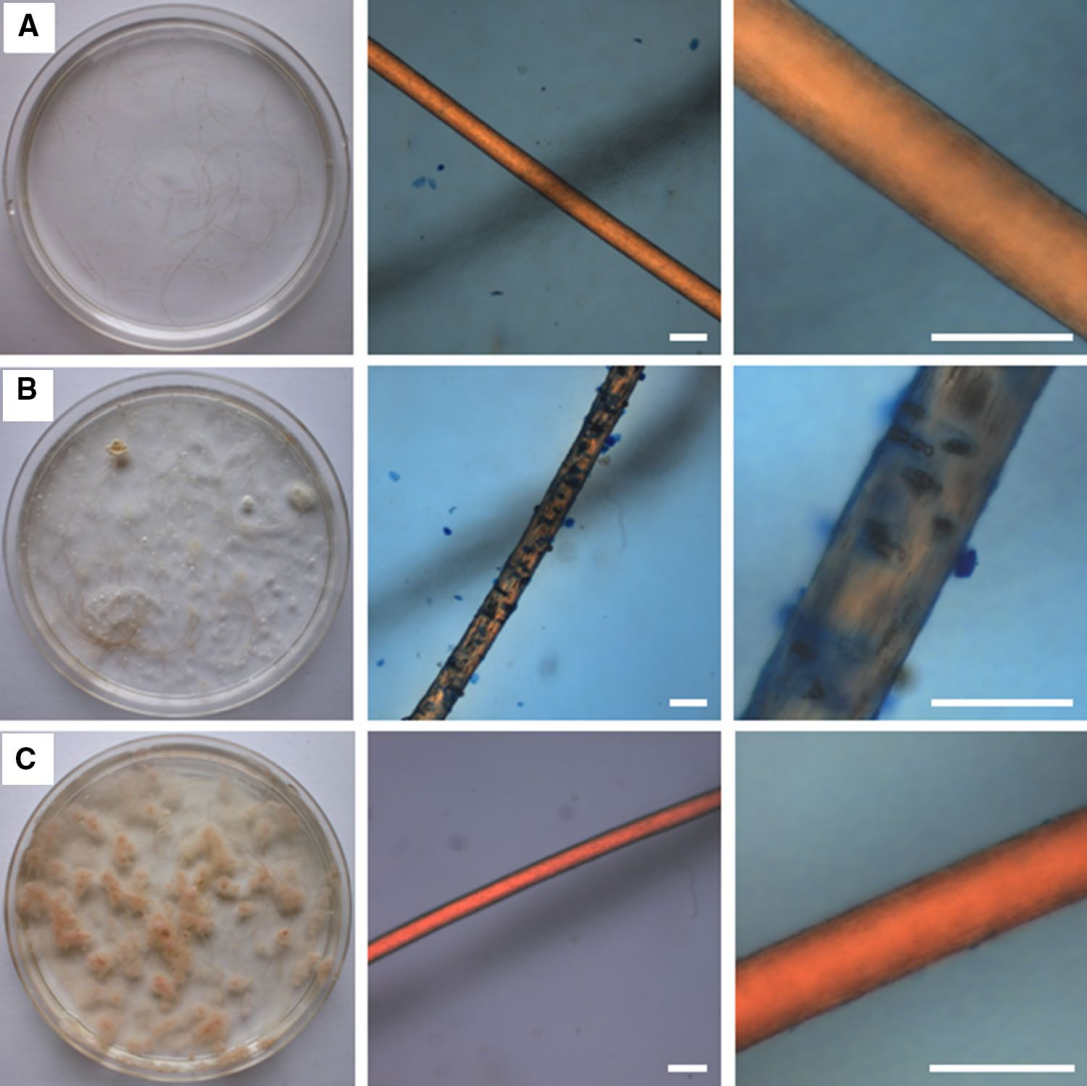
The in vitro hair perforation test (lactophenol cotton blue preparation): **a** negative control without fungi; **b** positive control with *Trichophyton mentagrophytes*; **c** *Penicillium glandicola* was negative. *Scale bars* 100 μm

The studied fungus showed differences in the ability to secrete extracellular enzymes. No pectinolytic and keratinophilic activity was detected. The most intensively synthesized enzymes were amylases, for which average enzymatic units (EU) in 1 mL of medium numbered 3.54 × 10^−3^ EU per 1 mL. On the other hand, the least intensively synthesized enzymes were cellulases—3.10 × 10^−5^ EU per 1 mL of medium (Figs. 6, 7).

**Fig. 8:**
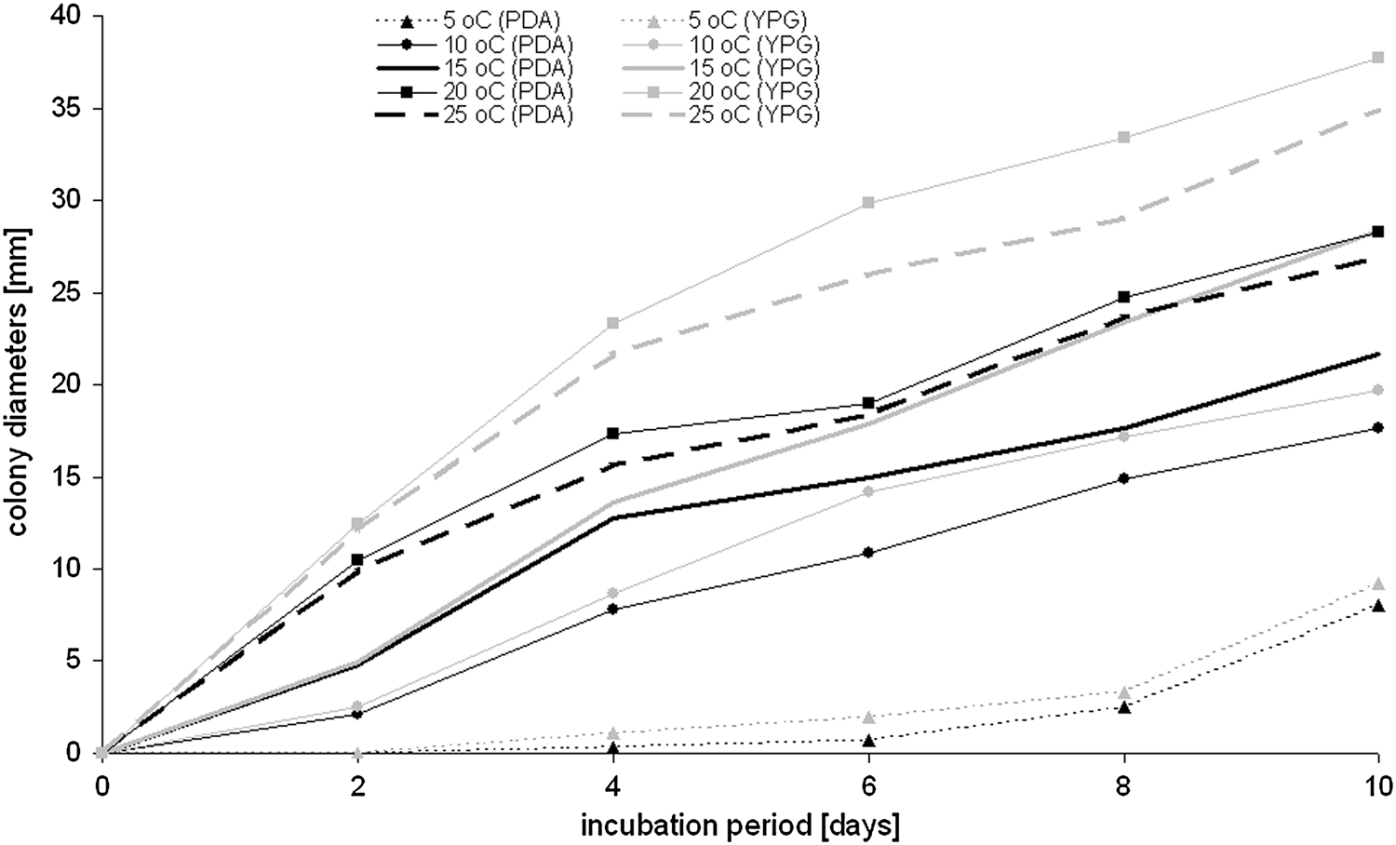
Average colony diameters of *Penicillium glandicola* at different temperatures on PDA and YPG, without active growth at 30 and 37 °C

Fourteen-day-old cultures of tested fungus cultured on PDA and YPG slants were able to survive for 56 days (the maximum time tested) at different temperatures, from −72 to 5 °C (Table 1). Additionally, the spores of this species were able to germinate and actively grow on PDA and YPG media at temperatures from 5 to 25 °C, but lacked this ability at 30 and 37 °C—Fig. 8. The optimal temperature for the growth of this isolate was 20 °C on both media. However, the colonies on YPG medium at all temperatures were larger (mean ca. 3.2 mm) than on PDA medium. In the case of both media, the fungus showed visible growth of colonies after 2 days of incubation at 10, 15, 20, and 25 °C, and after 4 days at 5 °C.

## Discussion

Fungi are eukaryotic microorganisms, comprising at least 1.5 million species, but only a few species are able to actively grow in underground niches. For example, white fungal stains consisting of *Fusarium solani* and black stains consisting of several filamentous fungi such as *Ochroconis lascauxensis*, *O. anomala*, *Acremonium nepalense*, *Scolecobasidium tshawytschae*, *Herpotrichiellaceae* sp. and *Verticillium* spp. and black yeasts such as *Exophiala moniliae* and *E. castellani* were found on the rock surface in the Lascaux Cave (Allemand and Bahn 2005; Bastian and Alabouvette 2009; Saiz-Jimenez et al. 2012; Martin-Sanchez et al. 2011, 2012a, b). We isolated only one fungal species (*Penicillium glandicola*) from the black stains on the surface of the clayey sediments on the wall in Driny Cave.

*Penicillium glandicola* (Oudem.) Seifert & Samson (often called *P. granulatum* Bainier) belong to Subgenus *Penicillium*, Section *Penicillium*, Series *Claviformia* according to the recent classification of Frisvad and Samson (2004). Literature reports about multiple varieties of *P. glandicola*, e.g., var. *confertum* Frisvad, Filt. & Wicklow, var*. mononematosum* Frisvad, Filt. & Wicklow, var. *glaucovenetum* Frisvad and var. *glandicola*. Our species is likely *P. glandicola* var. *glandicola*, because it was not able to grow at 30 and 37 °C, and it produces very rough stipes and branches. For example, *P. glandicola* var. *confertum* (syn. *P. confertum* (Frisvad, Filt. & Wicklow) Frisvad) showed very good growth at 37 °C (Frisvad and Samson 2004). On the other hand, *P. glandicola* var. *mononematosum* (as var. ‘*mononematosa*’, syn. *P. mononematosum* (Frisvad, Filt. & Wicklow) Frisvad), showed very good growth at 30 °C, but no growth at 37 °C, and *P. glandicola* var. *glaucovenetum* (syn. *P. concentricum* Samson, Stolk and Hadlok) no growth at 30 and 37 °C (Bridge 1985; Frisvad and Samson 2004). However, *P. glandicola* var. *glandicola* produce very rough stipes in contrast to var. *mononematosum* and var. *glaucovenetum* (Frisvad and Samson 2004).

This species may be isolated from a wide range of substrate types such as soil, foods, plants, air, bat guano, mammalian dung, earthworm casts, rock surfaces, etc. (Nováková 2009; Bezerra et al. 2012; Ogórek et al. 2016a, b). The literature also reported that the active growth of this species was found on marten excrement in Domica Cave (Slovakia), but it was identified only by morphological features and without detailed characterization (Nováková 2005). Therefore, our study is the first report regarding the active growth of *P. glandicola* in underground habitats supported with morphological and genetic identification and supplemented by detailed phenotypic and physiological characteristics of this strain.

Caves and other underground structures are likely a source of microbial extremophiles due to the extreme environments for life. Additionally, they provide ecological niches for highly specialized microorganisms (Schabereiter-Gurtner et al. 2004). Extremophiles are able to actively grow and survive in these environments, because they have developed mechanisms that allow them to cope with a variety of stressors (McKenzie et al. 2003). *Penicillium glandicola* isolated from the dark stains in Driny Cave was able to survive in vitro for 56 days at various low temperatures from −72 to 5 °C, and it was also capable of active growth in vitro on different media from 5 to 25 °C (optimal 20 °C). The obtained results correspond to reports of Frisvad and Samson (2004), in which *P. glandicola* is described as a psychrotolerant species that has growth optima in the range of mesophilic organisms, but is able to grow at low temperatures with much lower rates (Morita 1975; Wynn-Williams 1990).

Enzymes secreted by fungi play an important role in overcoming the natural resistance of the host (pathogenic process) as well as in providing soluble products that can be absorbed and used as food (Dobinson et al. 1996; Pekkarinen et al. 2000). *Penicillium glandicola* used in the study synthesized protease, cellulase and amylase, but not pectinase and keratinase. Therefore, this species is probably non-pathogenic to plants or humans and animals. Because pectinases cause modification of cell wall structure, increasing accessibility of cell wall components for degradation by other enzymes, cell lysis and plant tissue maceration (Panda et al. 2004). Thus, these enzymes are the first produced in infected tissue (Martínez et al. 1991). On the other hand, particularly important is the keratinophilic activity for the groups of fungi causing superficial fungal infections, e.g., dermatophytes that colonize skin, hair, and nails on the living host (Al-Fakih 2014). Because keratinous material is water insoluble and extremely resistant to degradation by common proteolytic enzymes such as trypsin, papain and pepsin, it has to be degraded by keratinases (Gupta and Ramnani 2006). Additionally, this species was not able to grow at 37 °C—this is a virulence factor for fungi that invade deep tissue of humans and animals, and the transition to the parasitic form is essential for the pathogenicity of dimorphic fungi (Tomee and Kauffman 2000).

Ogórek et al. (2016c) carried out a mycological research in Driny Cave at the same time as our study. They detected the spores of *P. glandicola* in the air and the rock surfaces inside the cave, but not detected their in the external environment of it. Thus, this species was not transferred from the external environment into the cave. Literature reports that fungal species belonging to the *Penicillium* genus can affect rocks through biochemical degradation by the secretion of organic acids, cause oxidation of Fe(II) and Mn(II), adsorption of Al, Zn, Cd, U, Th, Pb, and Sn, solubilization of rock phosphate and coal, reduction of Fe(III) as well as mineralization of materials such as halloysite and montmorillonite or todorokite (Sterflinger 2000; Burford et al. 2003; Cwalina 2008). However, there are no reports of *P. glandicola* that can affect rocks. Probably, this species benefited from organic matter which was transferred from the external environment, e.g., with sediment or water. According to Ogórek et al. (2016a), *P. granulatum* (syn. *P. glandicola*) was the most frequently isolated fungal species from the guano of bats and the air around it. Thus, this species may be associated with bats and guano. Currently, we are unable to accurately explain the role of this species in the occupied ecological niche. Therefore, we are also going to perform further studies for in-depth characterization of *P. glandicola* from Driny Cave.

## Conclusions

This is the first report regarding the active growth of *P. glandicola* in underground habitats with morphological and genetic identification and with the determination of phenotypic and physiological characteristics of this species. This species was able to synthesize amylases, proteases and cellulases, but not pectinases and keratinases. It was also capable of survival for 56 days at a broad range of very low temperatures (from −72 to 5 °C), active growth at temperatures from 5 to 25 °C, but without spore germination, and without active growth at 30 and 37 °C. Our research has shown that *P. glandicola* var. *glandicola* is a psychrotolerant species, which is capable of active growth under cold conditions as are typical for caves. The in vitro abilities of *P. glandicola* to produce extracellular enzymes and the lack of growth at 30 and 37 °C do not allow this fungus to be classified within pathogens that invade the surfaces and deep tissues of mammals or plants. This research contributes to our understanding of cave ecosystem, in particular to characterize the underground mycobiota and their role in the occupied ecological niche. Therefore, in the near future, we are also going to perform further studies for in-depth characterization of *P. glandicola* from Driny Cave.
